# The Effects of Demand Characteristics on Research Participant Behaviours in Non-Laboratory Settings: A Systematic Review

**DOI:** 10.1371/journal.pone.0039116

**Published:** 2012-06-19

**Authors:** Jim McCambridge, Marijn de Bruin, John Witton

**Affiliations:** 1 Faculty of Public Health & Policy, London School of Hygiene & Tropical Medicine, London, United Kingdom; 2 Department of Communication Science, Wageningen University, Wageningen, The Netherlands; 3 National Addiction Centre, King’s College London, London, United Kingdom; Tehran University of Medical Sciences, Iran (Islamic Republic Of)

## Abstract

**Background:**

The concept of demand characteristics, which involves research participants being aware of what the researcher is investigating, is well known and widely used within psychology, particularly in laboratory-based studies. Studies of this phenomenon may make a useful contribution to broader consideration of the effects of taking part in research on participant behaviour. This systematic review seeks to summarise data from studies of the effects of demand characteristics on participant behaviours in non-laboratory settings.

**Methodology/Principal Findings:**

Electronic databases were searched to identify eligible studies. These had to be purposely designed to evaluate possible effects of demand characteristics on at least one behavioural outcome under the autonomous control of the participants and use longitudinal study designs. Only 7 studies were included, 6 providing observational data and 1 experimental study, with 5 studies involving examination of possible effects on health behaviours. Although studies provided some evidence of effects of demand characteristics on participant behaviour, heterogeneous operationalisation of the construct, the limited number of studies and poor quality of study designs made synthesis and interpretation of study findings challenging.

**Conclusions/Significance:**

Although widely accepted as important in psychology, there have been few dedicated studies of the effects of demand characteristics on research participant behaviours outside laboratory settings. This body of literature does not currently contribute to the wider study of research participation effects. A systematic review of data from laboratory-based studies is needed, as are high-quality primary studies in non-laboratory settings. We suggest that unqualified use of the term demand characteristics should be abandoned.

## Introduction

The concept of “demand characteristics”, originating in the work of Martin Orne, is a little over 50 years old [1], [2]. It refers to participants being aware of what the researcher is trying to investigate, or anticipates finding, and what this implies for how participants are expected to behave. The concept is well known and widely used within psychology, but not in other disciplines. Orne was originally particularly struck by how helpful were research participants. He identified that research participants believed in the importance of science and tried to help advance it by playing the role of the “good subject”, thereby seeking to satisfy the perceived needs of the researchers [1], [2]. In these original accounts demand characteristics referred to the ways in which study participants responded to researchers, according to perceptions of their implicit preferences rather than their explicit instructions, within the specific context of the laboratory experiment. More recently, Orne has defined demand characteristics as “the totality of cues and mutual expectations which inhere in a social context…which serve to influence the behaviour and/or self-reported experience of the research receiver” [3].

Within a decade or so of their introduction to the literature the construct of demand characteristics was a well established feature of social psychology research (see for example [4]) and in experimental, psychology laboratory work in particular [5], [6]. The construct has since found its way into basic textbooks, though this does not mean that it has been uncritically accepted, however, as dedicated laboratory-based evaluation studies of demand characteristics manipulations show mixed findings [7]. The importance of differing research contexts to demand characteristics has been emphasised [8], as have specific implications for self-reported data [9], [10]. Evaluation of demand characteristics in the laboratory environment has evolved to be highly complex, as befitting the object of enquiry [11]. In laboratory-based studies, it remains common to deceive research participants about the true purpose of the study with blinding or distraction in order to prevent demand characteristics introducing unwanted influences on responses. This issue appears *prima facie* less well considered in relation to studies undertaken in other settings. It may be that demand characteristics are very different in the highly artificial situation of the laboratory as compared to non-laboratory settings.

Demand characteristics may be seen as one conceptualisation within a wider class of research participation effects, which historically have also been conceptualised in other ways, for example as the Hawthorne effect [12]. In addition to the implications of taking part in research studies per se, specific features of research participation have also been the subject of more focussed studies on for example the question-behaviour or mere measurement effect [13], or on aspects of the design of trials [14]. The position of demand characteristics within broader thinking about the psychological effects of research participation remains to be established.

The present work is motivated by a concern that demand characteristics may have particular impact in applied behavioural studies, where preventive measures such as blinding are difficult or often impossible to implement [15]. There have been no systematic reviews of the evidence on demand characteristics. The present study thus seeks to summarise data from studies of the effects of demand characteristics on participant behaviours in non-laboratory settings. Behavioural outcomes may potentially be objectively ascertained or self-reported and it may be instructive to compare the strength of evidence for each type of data [16]. An inclusive orientation is otherwise indicated, so restrictions on participants, interventions, comparisons and study designs will be avoided. The present study thus aims to synthesize existing evaluations of demand characteristics, examine how researchers have operationalized the construct, and draw conclusions about whether and under what conditions demand characteristics exist and may pose threats to the validity of results in applied behavioural studies.

## Methods

This is a systematic review reported according to the PRISMA statement [17]. Using this approach will elucidate whether the study of demand characteristics has been advanced in ways congruent with, and can inform, investigation of the wider consequences of research for the behaviour of participants in non-laboratory studies. Post-hoc references to, and discussions of, demand characteristics as possible explanations for observed data are widespread though probably not particularly informative for our purposes. We selected studies for inclusion in this systematic review according to the following criteria: 1. Used the term “demand characteristics” in the title or abstract. Where there was no abstract, the paper was required to include it in the introductory section, prior to the methods. 2. Peer reviewed journal papers only. Dissertations and other grey literature were excluded. 3. English language only (due to study constraints). 4. Empirical research reports only. 5. Non-laboratory settings. Where studies involved laboratory activity, they were required to also have a non-laboratory component. 6. Study design longitudinal in nature, as some time is required to elapse before demand characteristics may influence subsequent behaviour. 7. Studies must have included at least one behavioural outcome measure which is under the autonomous control of the research participant i.e. not test performance or evaluation of skills acquisition. 8. Studies must have been purposively designed to evaluate demand characteristics, defined broadly as involving an examination of any effects of research study participation itself. Overt instructions to alter behaviour or other interventions intended to change behaviour are excluded.

Studies of the effects of instructions to change behaviour are excluded because the researcher preferences are explicit, making this equivalent to an intervention e.g. [18]. Such an exclusion does not extend to experimental manipulations of demand characteristics themselves. It should be noted that the penultimate criterion omits a large literature comprising studies with non-behavioural outcomes such as effects on affect or cognitions e.g. [19], [20].

**Figure 1:**
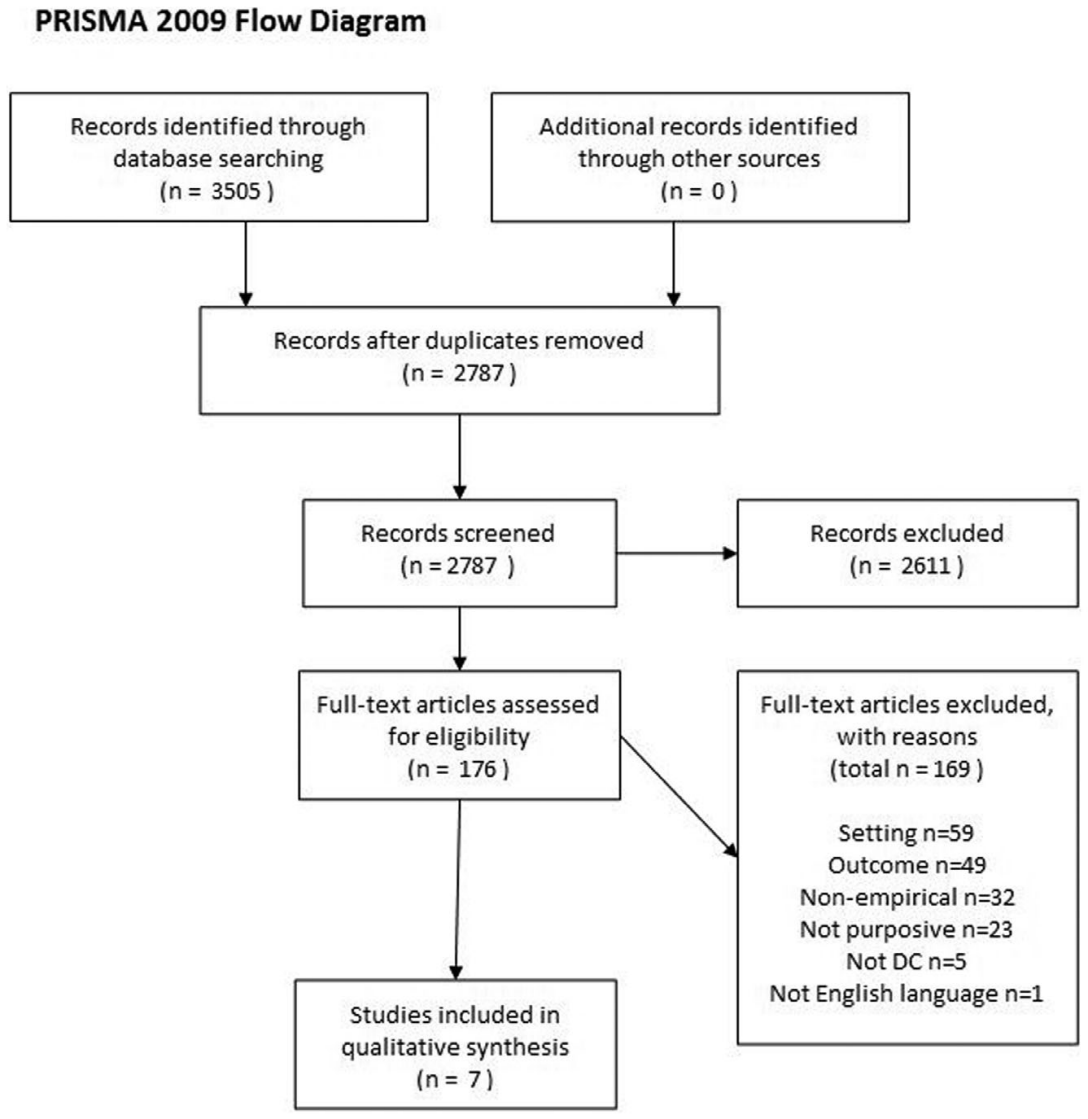
PRISMA flowchart.

The search strategy has been prescribed by criterion 1 above, which was used as a keyword search term. Electronic data base searching was undertaken in Web of Science (1970-), Medline (1950-), BIOSIS Previews (1969-), PsychInfo (1806-), CINAHL Plus with full text (1937-), ERIC (1966-), Pubmed (1950-), Cochrane Central Register of Controlled trials (undated), EMBASE (1947-), Sociological Abstracts (1952-), National Criminal Justice Reference Service Abstracts (NCJRS) (1970-), Social Services Abstracts (1979-), Linguistics and Language Behaviour Abstracts (LLBA) (1973-), the International Bibliography of the Social Sciences (IBSS) (1951-), APPI Journals (1844-), British Nursing Index (1992-), ADOLEC (1980-), Social Policy and Practice (1890-), British Humanities Index (1962-), Applied Social Sciences Index and Abstract (ASSIA) (1987-), INSPEC (1969-), PsychArticles (1988-). The most recent searches of all databases were undertaken on 11/12 January 2012. The 3,505 references obtained were reduced to 176 full text papers assessed for inclusion, as illustrated in the PRISMA flowchart in Figure 1. We did not identify any further studies for full text assessment through forward or backward searching. All eligibility decisions were made by two authors. We did not publish a protocol for this review.

Although it was viewed as being unlikely that data would be suitable for meta analysis as a result of heterogeneity in settings and behaviours permitted by the study design, no decisions were made *a priori* about the analysis of data in included studies. This expectation was borne out, and a narrative analysis of included studies is therefore presented with a table of basic summary data. This analysis incorporates content extracted by two authors describing the study, setting and context, the research questions being investigated, study design and nature of the behavioural outcome data, the detailed content of demand characteristics being evaluated, the study outcomes as reported and author conclusions, main likely sources of biases and the strength of the resulting evidence, along with comments on operationalisations or use of the demand characteristic construct.

## Results

Seven studies were eligible for inclusion in this review [21]–[27], as presented in Table 1. All but the earliest two are concerned with health behaviours. Five took place in the US and two in the UK. Two were studies of adolescents in specific institutional settings [22], [23], three were studies of university students [25]–[27] and two of adult populations in a workplace [21] and via newspaper recruitment [24] respectively. The early studies provided longitudinal data [21]–[23], followed by one experimental evaluation of demand characteristics [24], and three recent studies have assessed demand characteristics in studies nested within randomised controlled trials, also providing observational data [25]–[27].

**Table 1:** Included Studies.

Study	Study design	Study population &setting	Samplesize	Demand characteristicsbeing evaluated	Outcome data &outcomes as reported
Rosen (1970)^21^	Longitudinal beforeand after	Workers in US furniture manufacturing factory	73	The effects on productivity of simplymaking known the conduct of research	Productivity data, short livedproductivity increases
Kiley (1974)^22^	Longitudinal, before,during and after	Juvenile delinquents aged16–19 years old in a UScorrections unit	14	The effects of simply makingknown observation forresearch purposes	Unstructured activity assessedby observation. 3/4 behaviourschanged
Delamater (1988)^23^	Longitudinal studyof control groupwithin experiment	Adolescents with Type 1diabetes mellitus in a USoutpatient clinic	12	The effects of being given researchinstructions emphasizing honest,anonymous reporting	Self-reported blood glucoselevels increased
Faith (1998)^24^	Experimental	Adults recruited throughUS newspaper adverts	78	The consequences of measuringnegative affect-inducedeating	5-day food diaries to monitoreating, for which a sub-groupeffect found
Lewis (2007)^25^	Nested within RCT	US college students innormative feedback RCT	77	Whether normative feedbackgenerates ‘good subject’ biased self-reported outcomes on alcohol	No differences between trialfollow-up & apparentlyunrelated survey reports
Chapman (2009)^26^	Nested within RCT	UK college students inplanning interventionsRCT	212	Whether awareness of study aimsinfluenced self-reported outcomesfor healthy eating	Answers to open question codedaware or unaware. No differencesbetween-groups
Chapman (2010)^27^	Nested within RCT	As above	425	As above	As above

### Early Longitudinal Studies

In the earliest inclusion seven work groups with 8–10 employees each participated by completing two questionnaires. At time 1 the questionnaire assessed the preferences of each team member to be supervised by each of 7 foremen. One year later, researchers manipulated reassignments of foremen in order to examine whether preferences were linked to changes in productivity, as well as the effects of making known conduct of research [21]. At time 2, the conduct of the research study was revealed to the teams through completion of a second questionnaire, 10 weeks after the foreman reassignments. Short lived productivity increases were reported at both time points, during the ‘deception period’ and in the subsequent six week announced study period. These were stronger at time 2, leading to conclusions that observed outcomes were influenced by both the supervision changes and making known the research itself. The data presented for this effect, however, is not straightforward to interpret and would be judged weak by any contemporary standards, for example lacking statistical test results. The authors use the terms Hawthorne effect and demand characteristics inter-changeably to refer to awareness of being studied and related consequences for behaviour [21].

Kiley [22] similarly examined the possible impact of revealing to participants that they were being studied. Observations of 4 categories of behaviour were made over a period of 6 weeks: 2 weeks ‘pre-experimental’ 2 weeks ‘experimental’ during which participants were aware they were being observed “off and on throughout the day” and 2 weeks follow-up, after the participants were told the study was ended.

Three of the behaviours changed significantly during the declared observational period, and for two of these behaviours the effects were partially sustained during the follow-up period. The findings demonstrate notable changes in behaviours resulting from making known that behaviour was being observed. The author “cautioned for the possible contamination of the research outcome by the effects of the research process itself” [22]. ANOVA results suggest a large initial effect, and the ongoing effects after the participants believed the study had ended may well have resulted from lasting changes to interaction patterns, and thus not being problematic to the inferences made about the effects of being observed.

Delamater and colleagues [23] used an experimental design to compare low and high social demand characteristics where the latter involved intervention by clinical staff. This exclusion restricts attention here to change over time in the low demand conditionshowing increased self-reported mean blood glucose to a statistically significant degree in a one-tailed test one week later. [23]. Hence, this study offers modest evidence that expectancies about evaluation, influence either the reliability of self-report or actual adherence behaviours. The authors clearly believe the former explanation more likely, stating “This may be accounted for by our giving the subjects permission to report honestly with no threat of judgment or evaluation” [23].

### Experimental Evaluation of Demand Characteristics

Faith and colleagues [24] were concerned with the consequences of measuring negative affect-induced eating (NAIE) for both obese and non-obese adultswhere the experimental arm received a 10-minute lecture explaining that NAIE was associated with obesity while the control arm received no information. Participants came to a laboratory for questionnaire completion, taste test and lecture, and left with food diaries to monitor eating in the subsequent 5 days [24].

Unfortunately, since this was the sole experimental evaluation of demand characteristics included in this review, there is limited presentation of outcome data in this short report. Food diaries were completed by 78 participants (73% of those randomised). Outcome data were presented for a statistically significant interaction of study condition and weight, indicating greater NAIE with greater body mass in the experimental group compared to the control group Caution should be exercised in relation to this demand characteristics finding as it relates to a sub-group effect rather than a main effect. Data comparing those randomized to manipulation versus control were not presented.

### Studies Nested within Trials

Lewis and Neighbors [25] undertook a three-arm RCT in which U.S. college students (n = 185) were randomly assigned to either of two personalised normative feedback groups or to an assessment-only control group, with a one month follow-up assessment on a computer in a laboratory setting (n = 165 retained).

Forty-five percent of the total sample (n = 77) were selected for a sub-study when contacted by telephone 2 weeks after the post-intervention assessment. This involved an apparently unrelated survey of tailgating parties at football stadiums and alcohol use which was a topical issue for the campus at that time. Alcohol consumption was assessed in the same way as had been done in the trial. There were no differences between the two reports of the two drinking measures evaluated, neither across the groups as a whole nor between groups [25]. The authors concluded that demand characteristics had not interfered with trial outcome data. Only brief details of the data themselves are provided in this nested study (test statistics and information that p-values were not statistically significant), so it is not possible to assess directly the outcome data beyond a summary that there were no clear differences in this small sample. In this study it is also not possible to evaluate the safety of the conclusions drawn beyond making the observation that this design permits limited capacity to evaluate demand characteristics.

In another study nested within a 6-arm trial among UK university students Chapman and colleagues [26] investigated the effectiveness of two different types of planning interventions and pre-intervention instructions in relation to fruit and vegetables consumption in comparison with a non-intervention control group. The sub-study evaluating demand characteristics assessed whether awareness of study aims influenced reported outcomes [26].

Students consented to participate in a study of ‘dietary habits’. Three-hundred participants (of 600 randomised) provided follow-up data whilst attending classes one week later by completing a questionnaire that included planning, socio-cognitive variables and behavioural measures. It ended with the item “We are interested in what people think while they’re completing questionnaires like this. In particular we’d like to know what you think are the main purposes of this study. Please write your answers below” [26]. As with the previous study, there were no obvious problems with the self-reported data among the 212 coded responses though there is little data reported to examine this finding directly and it is thus not possible to fully evaluate theeffects of attrition and other possible biases It should be borne in mind here and elsewhere that the primary study aims are quite distinct from those of the present study.

A further 6-arm trial by Chapman and Armitage [27] had a similar design and purpose and demand characteristics were assessed exactly in the way described in the previous study. Six hundred and fifty paper and pencil questionnaires were completed at baseline, with 417 (64%) and 383 (59%) completing follow-up after 3 and 6 months, both by internet. Outcomes for those coded as not aware of the study aims at any time point (n = 367) were compared with those deemed aware (n = 58) and again there were no differences between randomised groups nor correlations with outcome at either follow-up point. These findings were again interpreted as meaning that reported increases in fruit and vegetable intake were not related to demand characteristics [27].

## Discussion

There have been few dedicated studies of the effects of demand characteristics on research participant behaviours outside laboratory settings. The older dedicated studies have weak study designs by contemporary standards and small sample sizes. The more recent studies nested within randomised trials have been designed to address secondary study aims with observational data. All but one of the included studies used non-experimental designs to evaluate the presence of demand characteristics as defined here. There is thus an absence of high quality experimental data on non-laboratory manipulations of demand characteristics. Little can be securely known about the effects of demand characteristics on participant behaviours across these studies as a whole. Diverse definitions of what constitutes demand characteristics have been used, ranging from awareness of conduct of research or being watched and their effects on actual behaviour, to reporting artefacts or some combination of both. It should be pointed out, however, that this diversity is in keeping with the most recent definition of demand characteristics offered by the original author, Orne [3].

Whilst falling outside the parameters of the present systematic review, it is not clear that there is any obvious body of laboratory-based review work on the subject of demand characteristics, despite the origins of the construct. The primary laboratory studies located in the course of the present work typically contained information on a small body of prior laboratory work in the immediate subject area. A systematic review is needed before drawing any conclusions about the utility of the construct in the laboratory setting. Notwithstanding this caution, this study calls into question whether the demand characteristics construct is useful for wider research purposes. The meagre extent of non-laboratory dedicated studies over a period of approximately 50 years was a surprise, particularly in light of the widespread use of this term in the literature. It is almost as if the construct has been accepted without being thoroughly interrogated in empirical investigations.

The term demand characteristics has also been applied within the literature to expectancies associated with various forms of interventions rather than to those pertaining to research per se. For example, Kanter et al. [28] distinguished experimental from psychotherapeutic demand characteristics, referring to the latter as the sum total of cues that convey the therapist's wishes, expectations, and worldviews to clients. Horvath [29] identified this type of expectancy of change to be an important component of common factors in psychotherapy. Moos and colleagues [30], [31] have similarly studied settings as generating demand characteristics in relation to change. It should be noted that such applications fall outside the interest in the demand characteristics of research studies examined here.

The definition of demand characteristics used in this study excluded direct instructions and co-interventions. To have included such studies would preclude clear determination of the existence of demand characteristics and the size of their effects because of confounding with other content. We restricted inclusion to studies including the term demand characteristics in the title or abstract and it is possible if not likely that studies which otherwise would meet inclusion criteria were missed. This decision was taken partly for practical reasons to reduce considerably the volume of screening to be done. As the term is so widely used, it seemed likely that few purposive studies (another of the selection criteria) would not use the term in either title or abstract. Nonetheless it must be acknowledged that any studies not successfully identified could alter study findings. In this regard, another study limitation to consider is the restriction to peer-reviewed literature. Exclusion of the grey literature may have omitted studies which found no effects of demand characteristics which were not submitted for publication as a consequence. This selection criteria decision was taken due to the likely difficulties involved in systematically searching the grey literature. Publication bias poses profound threats to the safety of inferences about effects in systematic reviews [32] and it is thus warranted to recognise the possibility that included studies over-estimate the true effects investigated here. We did not find any qualitative studies, though the selection criteria also required that they be longitudinal in nature, constituting another study limitation.

It is clear that dedicated studies of non-laboratory applications of demand characteristics have not produced a body of work that can contribute substantially to the wider study of research participation effects. Findings from some studies included here suggest that there are various research artefacts that are engendered through the formation of expectancies about researcher intentions and as a product of role performance as a research subject. The effects of research participation reported in the older studies should be considered alongside findings of the absence of obvious reporting bias in the three recent studies. The phenomena implicated here could also be labelled differently and may be more usefully conceptualised and studied in other ways. Alongside the present study other systematic reviews have been undertaken which explore other aspects of research participation effects as they have been previously evaluated (e.g. [33]. Perhaps the time is ripe or overdue for genuinely multi-disciplinary studies of these phenomena.

The authors expect that the study of research-based expectancies does have an important part to play in better understanding research data, and so frame this suggestion as an invitation to further study. It will not, however, be useful to permit a broad array of demand characteristics phenomena to be the object of such study, without clearly specifying their content. Operationalisation of what exactly is meant by this term is a necessary foundation for any careful experimental manipulation. Qualitative studies too will be very useful given the nature of the object of enquiry. We suggest that unqualified use of the term demand characteristics is not only questionable but should be abandoned, at least outside the laboratory. The widespread currency of the term within psychology, as indicated by its prominence within textbooks should perhaps not be discouraged. It should, however, not be used to describe phenomena as already well understood but rather as an important set of issues calling for a clearer understanding.
